# Laparoscopic Cholecystectomy Combined Using Miniaturised Instruments in Transgastric Gall Bladder Removal: Performed on 63 Patients

**DOI:** 10.1155/2010/582763

**Published:** 2010-01-28

**Authors:** Florent Jurczak, Jean-Paul Pousset

**Affiliations:** Department of General Surgery, Polyclinique de l'Océan, 38 rue de Pornichet, 44600 Saint Nazaire, France

## Abstract

*Background*. The laparoscopic cholecystectomy is a perfectly codified surgical procedure. The development of recent innovative and experimental surgical techniques Natural Orifice transluminal endoscopic surger (N.O.T.E.S.) which reduces the abdominal wall trauma leads us to develop a combined procedure of a standard dissection using miniaturised instruments already existing on the market (3 and 5 mm wide) and a gall bladder removal through a short gastrotomy Natural Orifice Specimen Extraction (N.O.S.E.). *Methods*. Our objective was to evaluate the safety, the feasibility, and the reproducibility of our new approach. After reviewing existing products on the market and a feasibility study, we put in place a protocol in our structure for patients on whom the procedure was performed. We carried out a gall bladder removal by a short gastrotomy, located on the anterior gastric wall, which then reduced the abdominal wall trauma and allowed them to resume normal physical activity quickly without risk of trocar site hernia. *Results*. We performed the procedure described in this paper on 63 patients, between April 2008 and July 2009. There were 14 men and 49 women with an average age of 46.8 years (ranging from 28 to 77) and an average BMI of 27.2. 30 patients had at least one gallstone larger than 10 mm. There was no postoperative gastric or abdominal wall complication and a fast recovery for all the patients in our study. *Conclusions*. This procedure is feasible, reproducible, with good results and minimal abdominal wall trauma. It is also safer than N.O.T.E.S. and endoscopic clipping and recovery, allowing normal physical activity, fast and, without risk of incisional hernia.

## 1. Introduction

The laparoscopy became the main surgical technique for cholecystectomy with a rate of laparoscopic performances of about 99% for some French teams. The development of recent innovative and experimental surgical techniques (N.O.T.E.S.) [1–4] reduces the abdominal wall trauma and complication by using ports and removal of the gall bladder or any other abdominal organs. The risk of incisional hernia increases when using a 10 mm or more port [5, 6]. The removal of an inflamed gall bladder with or without very large gallstones usually requires enlarging one of the abdominal incisions. It will be closed of course, scrupulously [7, 8], but there is always a risk of complication (infection, bruising, incisional hernia [9–11]) and that is the same problem with the single port access surgery as it is also difficult to get back to normal physical activity quickly, without risk of trocar-site incisional hernia (especially for patients who practise sport, manual workers or if, for example, they have to look after a dependant relative).

How to improve results? The N.O.T.E.S. has stimulated the surgeons' ingeniosity and the engineering department of medical laboratories. Some innovative materials have been developed but it requires surgical procedure modification. The standardization of the laparoscopic cystic duct dissection resulted in a reduction of the rate of common bile duct injuries which lead us to develop a combined procedure of a standard dissection using existing miniaturised instruments (3,5 mm wide), a 5 mm wide angle optic, and a gall bladder removal with a short gastrotomy. These 3,5 mm wide instruments were used in pediatric surgery, so we just had to increase their length [7, 8]. Currently, the 5 mm wide angle optic allows us to have a very good operative view thanks to the latest camera generation on the market. The transgastric gall bladder removal and its lithiasis can be performed under gastroscopic control or to tie the infundibulum to a nasogastric tube. The gastric incision is stitched up without difficulty and is safer than the endoscopic approach (clips, loop, etc.) [4, 12].

Our procedure (N.O.S.E.) was offered to any patient who had a symptomatic gallstone without emergency, acute cholecystitis, or common bile duct lithiasis. Patients with ORL and oesogastric history were excluded from the study. We chose not to take into account: age, BMI, and unusual medical contraindications for laparoscopy. It was agreed with the patients that we would perform this procedure if we did not encounter any surgical difficulties. If needed, we performed a standard laparoscopic or laparotomic surgery.

## 2. Our Surgical Procedure

We use a Veress needle insufflation in the left hypochondrium.

 We first put the 5 mm optic port in place in the peri umbilical or para and sus umbilical side of the abdominal wall according to the surgeon's normal routine. 

 We put two or three, 3,5 mm ports in place on vision control in the left hypochondrium, in the right side of the abdominal wall and in the epigastrium if necessary. The left hypochondrium port could be changed for a 5 mm one if we decided to use pediclar clips.

We start with a standard cystic pediclar dissection and we always try to do a systematic per operative cholangiography as we usually do in our surgical team Groupe de Recherche et d'Etude en Chirurgie Coelioscopique de l'Ouest (GRECCO) (Coeliogrecco.com).

 We tie up the pediclar ducts with stitches or clips.

 We then carry out a monopolar electrodissection of the gall bladder.

 We can evacuate the gall bladder fluid to reduce its volume if necessary.

 We tie up the infundibulum with a 2/0 monofilament stitch.

 A gall bladder bipartition with another loop can sometimes be required to avoid the agglutination of the gall stones to the bottom as it could hamper its removal.

 We must locate the large nasogastric tube (16 to 20 French which is put in place at the beginning of the procedure) or the end of the gastroscope.

We then carry out a longitudinal or vertical gastrotomy on the anterior side of the lower part of the stomach (2 to 3 cm long). 

Afterwards we tie the gall bladder with a stitch at the end of the nasogastric tube or at the end of the gastroscope: gall bladder removal.

Hand sew the short gastrotomy with braided or monofilament absorbable suture (3–0 or 2–0; 26 mm  1/2 c needle). We put in place these sutures through the 5 mm port without difficulty. The gastrotomy was hand sewn by separated stitches or overcast seams.

We then carry out a peritoneal washing, exsufflation, and removal of the ports.

Next morning, the patient can resume eating without restriction and takes one capsule of Proton-Pump Inhibitor (IPP) for 10 days.

If necessary we prescribe paracetamol analgesics after the surgery.

The patient leaves our hospital after a usual 48-hour stay for this pathology.

## 3. Study

This procedure was performed on 63 patients from April 2008 to July 2009, during which period there were 4 failures of the complete procedure.

These 63 patients were 14 men and 49 women, with an average age of 46,7 years (ranging from 22 to 77) and an average BMI of 27,3 (ranging from 22 to 36).

Eight female patients had some abdominal surgical history. 

31 patients had at least one 10 mm wide lithiasis; 18 patients had at least one 15 mm wide lithiasis; 9 patients at least one 20 mm wide lithiasis. The widest lithiasis was 30 mm.

49 patients required the use of a second 5 mm port to put pediclar clips. On 14 occasions only, we used a single 5 mm port when we put a stitch.

The cholangiography was systematic.

On 10 occasions, we carried out a gall bladder bipartition when there was a large number of lithiasis.

The gastrotomy was stitched up 40 times with absorbable monofilament and 23 times with absorbable braided suture: 9 times these were separated stitch and 54 times overcast seams.

The procedure's average length was 50 minutes (ranging from 35 to 80 mn).

During this study, there were two per operative difficulties: a very short cystic duct and an acute cholecystitis. These two procedures were brought to a successful conclusion. There were four failures. One was the impossibility of placing the nasogastric tube into the stomach, two were due to the presence of very large gall stones (3.3 and 4 cm wide), and one patient had an acute macro multilithiasis cholecystitis and a right hepatic artery. We carried out a standard laparoscopic cholecystectomy.

No gastric or abdominal wall complication occured during the hospitalization or during the 6 weeks postoperative consultations.

The majority of the patients (61/63) could resume normal physical activity after leaving our hospital.

## 4. Discussion

The development of the laparoscopic surgery since the 1990s, allowed the abdominal wall trauma reduction. In 2007, 88 000 laparoscopic cholecystectomies were performed in France in opposition to 7770 laparotomies (PMSI French data). However, it is well known that all of the trocar site hernia occurred through large (> or = 10 mm) port defect. According to the literature, the overall incidence of trocar site hernias is expected to be around 1% [5, 6]. The fascia closure should be done when ports > or = to 10 mm have been employed. So we can reduce the rate of incisional hernia. Of course, the abdominal organ removal (as the gall bladder and its lithiasis) often requires enlarging the fascia incision with a high rate of incisional hernia, bruise, and infection [9–11]. This abdominal wall trauma, even limited, prevents the patients from resuming normal physical activity very quickly especially for patients who practise sport, manual workers, or for example, if they have to look after a dependant relative. We thought that to use miniaturised instruments for dissection with ports less than 5 mm could be more beneficial. So the risk of trocar site incisional hernia is almost zero and allows resuming normal activity upon hospital discharge. To the contrary of the Natural Orifice Transluminal Endoscopic Surgery (N.O.T.E.S.), our pediclar dissection is standardized and identical to the usual procedure without risk of increasing the rate of biliary complication (common in this kind of procedure).

The gastric wall usually heals very quickly and well. In this way, the simple laparoscopic closure becomes the reference procedure with peptic ulcer perforation [13] with an average rate of complication less than 1% in the context of a gastroduodenal pathology. We carry out a hand sewn gastrotomy surgical closure of greater quality than the endoscopic closure [3, 4, 12]. We prescribe a 10-day IPP treatment after this procedure. 

Our N.O.S.E. procedure is simple and reproducible, using existing miniaturised instruments that we have adapted to adult surgery (long 3 mm wide instruments). The 5 mm wide angle optics allows having a very good operative view equivalent to a usual 10 mm optic used in laparoscopic surgery. Narrower optics could be developed in the future. The length of this procedure is slightly longer than the usual technique due to the transgastric removal and the gastric closure.

In our experience, the after effects are very simple without early or late abdominal wall and gastric complications in the followup. The patients resumed normal activity quickly. The esthetic result is perfect with minute skin incisions. The risk of gastric complications is very low in the context of a nonpathological gastric wall but will be the object of a more accurate assessment.

There are some difficulties with the gall bladder removal in cholecystitis and very large gall stones (larger than 3 cm). It could be overcome by carrying out endoscopic control of the removal with or without a specific cover. This endoscopic control could also be interesting in the presence of a hiatus hernia, an oesophagitis, and so forth.

## 5. Conclusion

Our procedure (laparoscopic cholecystectomy using micro instruments and a 5 mm optic in a transgastric gall bladder removal) allows abdominal wall trauma reduction and to resume as soon as possible normal physical activity without risk of incisional hernia. The cystic pediclar dissection is a usual and standardized procedure. The hand sewn gastrostomy is safer than N.O.T.E.S. and endoscopic clipping. It could open the way to other transgastric abdominal organ removals.
